# Supplementary material for: Functional analysis of Collagen 17a1: A genetic modifier of junctional epidermolysis bullosa in mice
Source: PLoS One. 2023 Oct 5;18(10):e0292456. doi: 10.1371/journal.pone.0292456 (PMC10553217; doi:10.1371/journal.pone.0292456)
Supplement: S2 Table — (DOCX) [file pone.0292456.s002.docx]

4034 nucleotide donor used with TAL1 (lower case indicate EcoRI ends):

gcttcgaattcAGGCCTGATTAATCATTAATGATAATCAAGACATCTTCATCTCTATACTTGGCTTCCAGTCATACATTAATTGCAAAGAAGCTTCTAATTTGTTGTCTATGTGTTTAGCTTTCAAGGAACTTAGAGAAAACAAAAGTGCTTTGTCTGGTGTGTCCAGATTGAAGTGGTAGCTTTTAAAACTCTGCCTTTAGAGATCTCAGTGATCCATTGCTGGAAGCCAGGTCTAAACTACCACCAATCACCTCTTCCATTTGTGACAAACAGTGTTGCAATCAGACCTATGTAACCCGGATCCTTTTTTTAAACTAACTTTCTCACATGATTAAACATACATTAAAAAAGAAAAAAAAACTTTAATTGAAAATTTTACAGTTGAAATGATTTTTCATCTTACAGGTTACATACAAATGTTTTTAGACAGTGGAAAGTGCTTAAATAAAAACCAACTGTGCCCAGTCTTTTATGCCATTTCCTAAGTGCAGGATGGTGTGTGGAAGCCATGCTTCCTCAGCATCAGGAGTTCTGTGTCCTCTCTTTTCTCCCTTCCTTGCCCACTGCCCCTCTTGTCCTGTCTGGGTCTAGTGTGGTGTCATAATTGTTACTTCTGGCCTGTACCTGTGGACACAGGCCATAGCTACGACCAGTCAAGCTTTGTAAGTCAGTGGGAAGGTGATTGGCATCTGCACCATGGTAACTGAAATGTTACCTCCTCTAAGGAAGTGAACTTCTGAGTGTTTTCAGTTGCAAGAGTCACCTCTGTGACTGGAGAATGCAGAGTGAAGAGAGGAGGAAGGAGCTGACAGCAGCCTTCGTAACATAACCCATCACATGTCATCTACAAAGGCCCCCATTCCCATTTTCCATTTTCTGGGCTCACTTTTTAGGCAAAGCTCCAAGATCCCCAGCCAACAGTTATATAACTGGCTTCCGAGGCCTGGCAGAGGCCTGCGCTAAATGCTAAGGAAGCCAGGGAGCTCAGATCTATGTAGCAGAGAGGTCAAGTCTCATATTAAACATTGCAGCTATTCCGAAAGGCCCACCCAACCAATGGAGCTGTTTCAGATTGTGTGTCTTTGAATTTTCTGTATGTATATATTTGGTTCAGACTAAAACAAGTGTCGCTGGGTGATTTGGTCTCAGGAGGACGGGGACCCTTGGACATAGGCGCCCAAGAACCGGTCCCAGATACTTGGTGGCTCTGCCAAGTGCAGCTTACGGCTTGATGGCAATACTTCTCTTCCTTCTCCCAGTATAGACTTGGTCACCTGAAAGTCCAAAGATGAGCAGGGGTTAAATGCCTTGTGGCCTATGACCTTAGGGAGGACCTGCGATACCAACAGTGATTGCTAAAAGGTAGCCTGGAAGATTAATCCTTTGTAACGCTATTTTTTCCCTTTCAAGTCATCTCCCTACTTTGTTACCAGGTTCTTCCTCACTCTGCGGCAGCTCTGGTCTTCCCTTTTCCCCAGCTGTGCAACACCATTTTCTGCATTGGTTTTTGCTTGATTTTTCTCAGGCTAGCCCAGAACCAAATGCCCTCTTCTCTGCTGGATCCCAAAACCCCAGCAGTCACACACCACTGCTTACCTTTGTCTCCTTTCTCTCCTTTGTTCCCTCTGGGGCCGGGTGGCCCTGGAGGACCTCGTGGTCCAGCAGGGCCCCTGTCACCTAGAAGGACAGAGAGGGCATTGACACCAGAGTATGAGTACAGAGCTGAAGGCGTGGCACAGTGTGGCACTAAGGCATAAACAGCTGACCCTGGAGTCCCTGCTGTCACTTACCTTTGGGGCCTGGGGTTCCCACATCTCCCTTCTGCCCAGGTGGTCCCGGGATAGTGCCGTAGGCTGCAAGGAAGGAAAGAGTCTAGCCTTAGACCAGAGTTTGCCTGAGGCTTGGGAGGTCAAGCAACACAGAGAGTGGTGGCATCAGGACTTATACAAGGAACCAGAGGTAGGCTTGAGTTTTGGCATCTCAGCTATGGGGAGAAGATCATTACGTTGGTGTTTGTATGCCAGGCCCCACGAAAGACTAAGCAGCAGGCCAGTGCTGGGCAAGCCATGGAGCTATGCTTACTTTGGAAGAAGTCCATGAGGTCTTGTGTCACGTTGCTGTAGGCTGAGAAGACCCTGCTGATGCCAGGAGGGCCTTGGGGCCCAGGTGGGCCCTGGACAGTGTAGGCCATCCCTTGTAGCAGACCCTGACCTGCAGAATACCAGGGTGCTGGTGTAGGAAGCAGCATACCACTTCGCTGCCGAAGGGCAGTATCCTCGGACTGCCTCACTCAGAGCAGCTAAACTAGATGAAACCAGCCTGAGGGAAAAGCCCTGCAGCTTCCTTTGCTGTCCAGCAGACTGGGCTGACCTGTTGTGACAGTTCTCGGGGACATTAGGACTACAGGATGTGCTGGTAGCTAAAGTGAGAAAGGGCCTCCTGGCTAACAACTGCCACGAAGCCCTGTCAGAAAGCCGCTCCCATCAGACTTGTTCCGAGCAAAGTACCAAGCCCTTTGCTGTGTGCCCTGCTCTGCACAGCTCAGTGTCAGCACTGGGGTCCCTTACACTGAGAGAGTGGGGTCTTTTTCTCTTTCAATCCCCACCCATCTGATTCCAGAGCCGCTATCTACACAGTCCAAGTACATAAAAAAACGAGGGCCAGTTTTAGGCCTGCAAAGCCTAAATGCTGAGGTCCACACATGCTCCTTCCCCCTCTGAGGGCCACAGGTTCCAACTTACGCTGCATGCTCTCCGACACCCGCACTGCCAGCTTGTTGTAATCTAGGTCTCCAGTGAAGCCATCCCGGAACGAGTCACCATTGGTGCCATATATACCACCACCTGCAGCTGCCCCATAGCCTCCGCCTGGGCCGATGTCAGTACCATAGGGGCCCCCATCTCCTGCACCATAGGCTCCACCTTCACCCAGGGAGCCTCCGTTGGTTCCCCCTATGCCCGTGGAAGAGCTGTAGGAAGTGCCCCTTCTGGCTGAGGAGCTGCTGCCCCAGTTGTAGTTCTCCCTTAAGTGACCATCTCCAGGTGGTCCCTGTGGCCCCGGAGGGCCTGGAGGGCCAACGATGAAGCTGCGAACATCAGGACCTGTAGGGTAGGAAGATGCATTACCGGCAGACTGTGGTGGACAAAAGAGTTGGGAGCTAGGGGGGTCTCTGGAGTTTTAACACCCCTTAGAGTGACCTTTGAGGCCCTTGTACATGTACAGGATGCTGGTGAGCAGAGAGTGGTTTATCTAGACTGCTGACCATCCTGTGGCTCCTGCAGAAGAGTTGTGAAGGTCTAGGGGGAGGGCACAGGAGACCAACATCAAGGTACCTACTTGTGAGGTAGCTAATCAGCTCGCTGCGGAAGTTGTCACTGTTTTCAGCTGCATAGGTGGACAGAGCTGCTGAGACACCTGGGGGCCCTCGAGGACCTGGAGGCCCTGGTGGGCCTGGAGGGCCTGGGATAGAGGACAAACCAGCGGCTGTGTGAGAGGGGAGGGAAAGAGGAAGGTCAGGATCCTGGACAGAAAAGGCTAGATTCTCAGCCTGGCCTTGACCTACTGCGGGCCCCCTCACCCCCCATGGATTGGGTGCAAGAGATGGGTATGTGTGTGTGTGTGTGTGTGGAGTAGAGGAGAGTGGGCAAGGCCTAGGTTGTCCCTTTCAGGCGATATCTAGGAATGGCCGCTTTCAAGGGACCCTTCTCCCCTTCACCCCATACATATCCTATGTCCTTCTAGACCTTCATCACCAGGCATTAGAATGTAATACAAGGTATTACATTATGTCCTTGTTAAGTGACTGATCACATGGCTCATGAGCTCTGCAAAGGCTACAGTTTGGAGCTTAGTTTCTTCCCATTTCTTCCCCTACCAAGGCTCATGGCTCCTCACAGCCGTGTGACCCTGTCCAGGGCCCCAGGGCCCTCCTGGTGCATGCTCCACTGGGGAACAGCACATTGAATCAGTTCTCCATGGCAGCCCTAGAGAGGGCTGGGGATGCCCTCAAAGTCAGGCTGCAgaattcgcttc

200nt ssDNA donor for use with TAL7:

AGGGACCACCTGGAGATGGTCACTTAAGGGAGAACTACAACTGGAGCAGCAACTCCTCAGCCAGACAAGGCACTTCCTACAGCTCTTCTACCGGCACAGGGGGAACCAACGGAGGCTCCCTGGGTGAAGGTGGAGCCTATGGTGCAGGAGATGGGGGCCCCTATGGTACCGACATCGGCCCAGGCGGAGGCTATGGGGCA

Gray is where TALENs sit. Pink denotes changed nucleotides.

Inverse 200nt ssDNA donor, also used with TAL7:

TGCCCCATAGCCTCCGCCTGGGCCGATGTCGGTACCATAGGGGCCCCCATCTCCTGCACCATAGGCTCCACCTTCACCCAGGGAGCCTCCGTTGGTTCCCCCTGTGCCGGTAGAAGAGCTGTAGGAAGTGCCTTGTCTGGCTGAGGAGTTGCTGCTCCAGTTGTAGTTCTCCCTTAAGTGACCATCTCCAGGTGGTCCCT

53nt ssDNA donor #1358, used with TAL10:

TCACTTAAGGGAGAACTACAACTGGGGCAGCAGCTCCAGCGCCAGAAGGGGCA

Gray denotes where the vector aligns with the TALENs and pink the residues altered in the vector compared to B6 reference.

53nt ssDNA donor #1359, the inverse of 1358, also used with TAL10: TGCCCCTTCTGGCGCTGGAGCTGCTGCCCCAGTTGTAGTTCTCCCTTAAGTGA

D57 200nt ssDNA donor used with CRISPR/Cas9:

GCCTATGGTGCAGGAGATGGGGGCCCCTATGGTACCGACATCGGCCCAGGCGGAGGCTATGGGGCAGCTGCAGAAGGCGGCATGTATGCTGGCAATGGCGGACTATTGGGAGCTGACTTTGCTGGAGATCTAGATTACAACAAGCTGGCAGTGCGGGTGTCGGAGAGCATGCAGCGTAAGTTGGAACCTGTGGCCCTCAG

G57 200nt ssDNA donor used with CRISPR/Cas9:

GCCTATGGTGCAGGAGATGGGGGCCCCTATGGTACCGACATCGGCCCAGGCGGAGGCTATGGGGCAGCTGCAGAAGGCGGCATGTATGCTGGCAATGGCGGACTATTGGGAGCTGACTTTGCTGGAGGTCTAGATTACAACAAGCTGGCAGTGCGGGTGTCGGAGAGCATGCAGCGTAAGTTGGAACCTGTGGCCCTCAG

Yellow highlight is 57nt region mismatched to B6 with red letters indicating specific mismatches. Green highlight is amino acid difference between D57 and G57.
