# Supplementary material for: Functional analysis of Collagen 17a1: A genetic modifier of junctional epidermolysis bullosa in mice
Source: PLoS One. 2023 Oct 5;18(10):e0292456. doi: 10.1371/journal.pone.0292456 (PMC10553217; doi:10.1371/journal.pone.0292456)
Supplement: S3 Table — (DOCX) [file pone.0292456.s003.docx]

**Table S3. Full TALEN sequences (Joung #s DR-TAL-0727-28) for intron 50 cut (TALEN1).**

Left TALEN1 (2325 bp):

TCTAGAGCTAGCACCATGGACTACAAAGACCATGACGGTGATTATAAAGATCATGACATCGATTACAAGGATGACGATGACAAGATGGCCCCCAAGAAGAAGAGGAAGGTGGGCATTCACCGCGGGGTACCTATGGTGGACTTGAGGACACTCGGTTATTCGCAACAGCAACAGGAGAAAATCAAGCCTAAGGTCAGGAGCACCGTCGCGCAACACCACGAGGCGCTTGTGGGGCATGGCTTCACTCATGCGCATATTGTCGCGCTTTCACAGCACCCTGCGGCGCTTGGGACGGTGGCTGTCAAATACCAAGATATGATTGCGGCCCTGCCCGAAGCCACGCACGAGGCAATTGTAGGGGTCGGTAAACAGTGGTCGGGAGCGCGAGCACTTGAGGCGCTGCTGACTGTGGCGGGTGAGCTTAGGGGGCCTCCGCTCCAGCTCGACACCGGGCAGCTGCTGAAGATCGCGAAGAGAGGGGGAGTAACAGCGGTAGAGGCAGTGCACGCCTGGCGCAATGCGCTCACCGGGGCCCCCTTGAACCTGACCCCAGACCAGGTAGTCGCAATCGCGAACAATAATGGGGGAAAGCAAGCCCTGGAAACCGTGCAAAGGTTGTTGCCGGTCCTTTGTCAAGACCACGGCCTTACACCGGAGCAAGTCGTGGCCATTGCAAGCAATGGGGGTGGCAAACAGGCTCTTGAGACGGTTCAGAGACTTCTCCCAGTTCTCTGTCAAGCCCACGGGCTGACTCCCGATCAAGTTGTAGCGATTGCGTCGCATGACGGAGGGAAACAAGCATTGGAGACTGTCCAACGGCTCCTTCCCGTGTTGTGTCAAGCCCACGGTTTGACGCCTGCACAAGTGGTCGCCATCGCCTCCAATATTGGCGGTAAGCAGGCGCTGGAAACAGTACAGCGCCTGCTGCCTGTACTGTGCCAGGATCATGGACTGACCCCAGACCAGGTAGTCGCAATCGCGAACAATAATGGGGGAAAGCAAGCCCTGGAAACCGTGCAAAGGTTGTTGCCGGTCCTTTGTCAAGACCACGGCCTTACACCGGAGCAAGTCGTGGCCATTGCATCCCACGACGGTGGCAAACAGGCTCTTGAGACGGTTCAGAGACTTCTCCCAGTTCTCTGTCAAGCCCACGGGCTGACTCCCGATCAAGTTGTAGCGATTGCGTCGAACATTGGAGGGAAACAAGCATTGGAGACTGTCCAACGGCTCCTTCCCGTGTTGTGTCAAGCCCACGGTTTGACGCCTGCACAAGTGGTCGCCATCGCCAGCCATGATGGCGGTAAGCAGGCGCTGGAAACAGTACAGCGCCTGCTGCCTGTACTGTGCCAGGATCATGGACTGACCCCAGACCAGGTAGTCGCAATCGCGTCAAACGGAGGGGGAAAGCAAGCCCTGGAAACCGTGCAAAGGTTGTTGCCGGTCCTTTGTCAAGACCACGGCCTTACACCGGAGCAAGTCGTGGCCATTGCAAATAATAACGGTGGCAAACAGGCTCTTGAGACGGTTCAGAGACTTCTCCCAGTTCTCTGTCAAGCCCACGGGCTGACTCCCGATCAAGTTGTAGCGATTGCGAATAACAATGGAGGGAAACAAGCATTGGAGACTGTCCAACGGCTCCTTCCCGTGTTGTGTCAAGCCCACGGTTTGACGCCTGCACAAGTGGTCGCCATCGCCAACAACAACGGCGGTAAGCAGGCGCTGGAAACAGTACAGCGCCTGCTGCCTGTACTGTGCCAGGATCATGGACTGACCCCAGACCAGGTAGTCGCAATCGCGAACAATAATGGGGGAAAGCAAGCCCTGGAAACCGTGCAAAGGTTGTTGCCGGTCCTTTGTCAAGACCACGGCCTTACACCGGAGCAAGTCGTGGCCATTGCAAGCAATGGGGGTGGCAAACAGGCTCTTGAGACGGTTCAGAGACTTCTCCCAGTTCTCTGTCAAGCCCACGGGCTGACTCCCGATCAAGTTGTAGCGATTGCGTCGCATGACGGAGGGAAACAAGCATTGGAGACTGTCCAACGGCTCCTTCCCGTGTTGTGTCAAGCCCACGGTTTGACGCCTGCACAAGTGGTCGCCATCGCCAGCCATGATGGCGGTAAGCAGGCGCTGGAAACAGTACAGCGCCTGCTGCCTGTACTGTGCCAGGATCATGGACTGACACCCGAACAGGTGGTCGCCATTGCTTCCCACGACGGAGGACGGCCAGCCTTGGAGTCCATCGTAGCCCAATTGTCCAGGCCCGATCCCGCGTTGGCTGCGTTAACGAATGACCATCTGGTGGCGTTGGCATGTCTTGGTGGATCC

Right TALEN1 (2325 bp):

TCTAGAGCTAGCACCATGGACTACAAAGACCATGACGGTGATTATAAAGATCATGACATCGATTACAAGGATGACGATGACAAGATGGCCCCCAAGAAGAAGAGGAAGGTGGGCATTCACCGCGGGGTACCTATGGTGGACTTGAGGACACTCGGTTATTCGCAACAGCAACAGGAGAAAATCAAGCCTAAGGTCAGGAGCACCGTCGCGCAACACCACGAGGCGCTTGTGGGGCATGGCTTCACTCATGCGCATATTGTCGCGCTTTCACAGCACCCTGCGGCGCTTGGGACGGTGGCTGTCAAATACCAAGATATGATTGCGGCCCTGCCCGAAGCCACGCACGAGGCAATTGTAGGGGTCGGTAAACAGTGGTCGGGAGCGCGAGCACTTGAGGCGCTGCTGACTGTGGCGGGTGAGCTTAGGGGGCCTCCGCTCCAGCTCGACACCGGGCAGCTGCTGAAGATCGCGAAGAGAGGGGGAGTAACAGCGGTAGAGGCAGTGCACGCCTGGCGCAATGCGCTCACCGGGGCCCCCTTGAACCTGACCCCAGACCAGGTAGTCGCAATCGCGTCAAACGGAGGGGGAAAGCAAGCCCTGGAAACCGTGCAAAGGTTGTTGCCGGTCCTTTGTCAAGACCACGGCCTTACACCGGAGCAAGTCGTGGCCATTGCAAATAATAACGGTGGCAAACAGGCTCTTGAGACGGTTCAGAGACTTCTCCCAGTTCTCTGTCAAGCCCACGGGCTGACTCCCGATCAAGTTGTAGCGATTGCGTCGAACATTGGAGGGAAACAAGCATTGGAGACTGTCCAACGGCTCCTTCCCGTGTTGTGTCAAGCCCACGGTTTGACGCCTGCACAAGTGGTCGCCATCGCCTCCAATATTGGCGGTAAGCAGGCGCTGGAAACAGTACAGCGCCTGCTGCCTGTACTGTGCCAGGATCATGGACTGACCCCAGACCAGGTAGTCGCAATCGCGTCGAACATTGGGGGAAAGCAAGCCCTGGAAACCGTGCAAAGGTTGTTGCCGGTCCTTTGTCAAGACCACGGCCTTACACCGGAGCAAGTCGTGGCCATTGCAAGCAACATCGGTGGCAAACAGGCTCTTGAGACGGTTCAGAGACTTCTCCCAGTTCTCTGTCAAGCCCACGGGCTGACTCCCGATCAAGTTGTAGCGATTGCGTCGAACATTGGAGGGAAACAAGCATTGGAGACTGTCCAACGGCTCCTTCCCGTGTTGTGTCAAGCCCACGGTTTGACGCCTGCACAAGTGGTCGCCATCGCCAACAACAACGGCGGTAAGCAGGCGCTGGAAACAGTACAGCGCCTGCTGCCTGTACTGTGCCAGGATCATGGACTGACCCCAGACCAGGTAGTCGCAATCGCGTCGAACATTGGGGGAAAGCAAGCCCTGGAAACCGTGCAAAGGTTGTTGCCGGTCCTTTGTCAAGACCACGGCCTTACACCGGAGCAAGTCGTGGCCATTGCAAATAATAACGGTGGCAAACAGGCTCTTGAGACGGTTCAGAGACTTCTCCCAGTTCTCTGTCAAGCCCACGGGCTGACTCCCGATCAAGTTGTAGCGATTGCGTCGAACATTGGAGGGAAACAAGCATTGGAGACTGTCCAACGGCTCCTTCCCGTGTTGTGTCAAGCCCACGGTTTGACGCCTGCACAAGTGGTCGCCATCGCCTCCAATATTGGCGGTAAGCAGGCGCTGGAAACAGTACAGCGCCTGCTGCCTGTACTGTGCCAGGATCATGGACTGACCCCAGACCAGGTAGTCGCAATCGCGAACAATAATGGGGGAAAGCAAGCCCTGGAAACCGTGCAAAGGTTGTTGCCGGTCCTTTGTCAAGACCACGGCCTTACACCGGAGCAAGTCGTGGCCATTGCAAGCAACATCGGTGGCAAACAGGCTCTTGAGACGGTTCAGAGACTTCTCCCAGTTCTCTGTCAAGCCCACGGGCTGACTCCCGATCAAGTTGTAGCGATTGCGTCGCATGACGGAGGGAAACAAGCATTGGAGACTGTCCAACGGCTCCTTCCCGTGTTGTGTCAAGCCCACGGTTTGACGCCTGCACAAGTGGTCGCCATCGCCAGCCATGATGGCGGTAAGCAGGCGCTGGAAACAGTACAGCGCCTGCTGCCTGTACTGTGCCAGGATCATGGACTGACACCCGAACAGGTGGTCGCCATTGCTTCCCACGACGGAGGACGGCCAGCCTTGGAGTCCATCGTAGCCCAATTGTCCAGGCCCGATCCCGCGTTGGCTGCGTTAACGAATGACCATCTGGTGGCGTTGGCATGTCTTGGTGGATCC
