# Supplementary figures and images for: Functional analysis of Collagen 17a1: A genetic modifier of junctional epidermolysis bullosa in mice
Source: PLoS One. 2023 Oct 5;18(10):e0292456. doi: 10.1371/journal.pone.0292456 (PMC10553217; doi:10.1371/journal.pone.0292456)

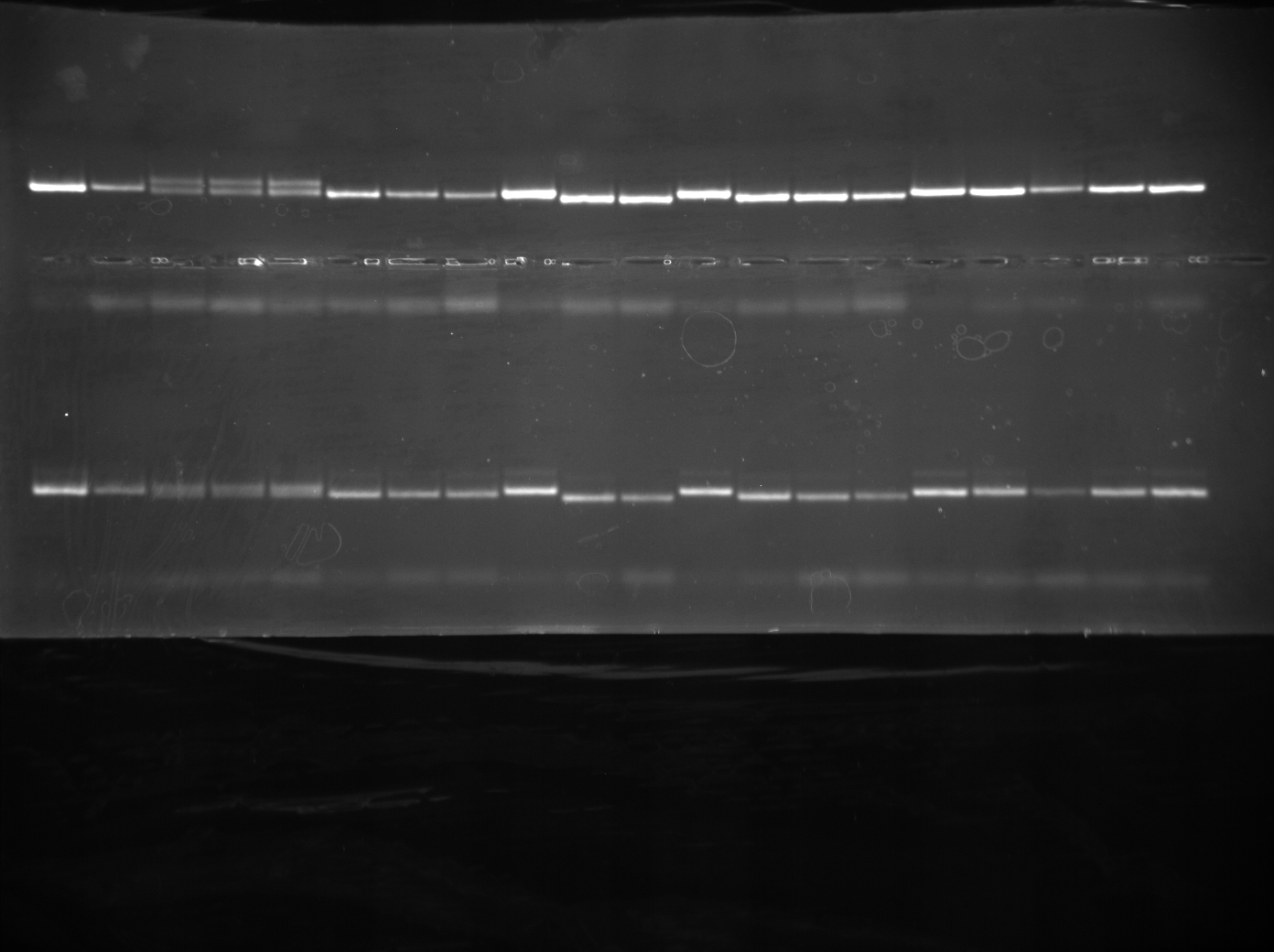

Supplement: S1 Fig — (BMP) [file pone.0292456.s005.bmp]
